# Prenatal Three-Dimensional Ultrasound Detection of Adducted Thumbs in X-Linked Hydrocephaly: Two Case Reports with Molecular Genetic Studies

**DOI:** 10.1155/2015/561713

**Published:** 2015-05-20

**Authors:** Edgardo Corral, Andres Barrios, Monica Isnard, Pascale Saugier-Veber, Sophie M. Fortier, Sarah Durrin, Waldo Sepulveda

**Affiliations:** ^1^Prenatal Ultrasound Unit, Department of Obstetrics and Gynecology, Regional Hospital, University Diego Portales, Rancagua, Chile; ^2^Pediatric Neurology Unit, Department of Pediatrics, Regional Hospital, Rancagua, Chile; ^3^Fetal Medicine Unit, Mulhouse Hospital, Mulhouse, France; ^4^Department of Genetics, Rouen University Hospital, Rouen, France; ^5^Fetalmed-Maternal-Fetal Diagnostic Center, Las Condes, Santiago, Chile

## Abstract

X-linked hydrocephaly is a rare sex-linked genetic recessive condition occurring in 1/30,000 deliveries. Adduction of thumbs and mental retardation are additional associated clinical findings. We describe two cases of X-linked hydrocephaly with associated adducted thumbs that were diagnosed prenatally with the combined use of three-dimensional (3D) ultrasound and fetal blood sampling for cytogenetic and molecular analyses. This report suggests that 3D ultrasound can facilitate the identification of adducted thumbs in fetuses affected by X-linked hydrocephaly and supports evaluation of the fetal hands as an integral part of the ultrasound anatomical assessment in male fetuses with hydrocephaly secondary to aqueductal stenosis.

## 1. Introduction

X-linked hydrocephaly is a rare sex-linked genetic recessive condition occurring in 1/30,000 deliveries. Bickers and Adams [1] first termed it hereditary familial hydrocephaly when they described the condition in 1949 and identified aqueductal stenosis as the cause of hydrocephalus through postmortem studies. In 1961, Edwards [2] recognized both adduction of thumbs and mental retardation as additional associated clinical findings. Subsequent genetic studies in affected families suggested a mutation of the X-chromosome. Indeed, in 1992 Rosenthal et al. [3] were the first to identify a mutation of the L1 gene in members of a family affected by X-linked hydrocephaly.

Further investigation demonstrated that X-linked hydrocephaly has a wide phenotypic presentation and shares a common genetic origin with other forms of hydrocephaly, which has been grouped under the denomination of L1 syndrome. Recently, it was determined that this condition is caused by mutation in the exon of the L1CAM (L1 cell adhesion molecule) gene located in the subchromosomal region Xq28. Over 200 different mutations have been reported, one-third of them being missense mutations [4]. While the L1 syndrome includes a variety of conditions, the most prevalent are X-linked hydrocephaly with stenosis of the aqueduct of Sylvius (HSAS, OMIM #307000); mental retardation, aphasis, shuffling gait, adducted thumbs (MASA, OMIM #303350); X-linked spastic paraplegia 1 (SPG1); and X-linked agenesis of the corpus callosum [5].

In this report, we describe two cases of X-linked hydrocephaly with associated adducted thumbs that were diagnosed prenatally with the combined use of three-dimensional (3D) ultrasound and fetal blood sampling for cytogenetic and molecular analyses.

## 2. Case 1

**Figure 1:**
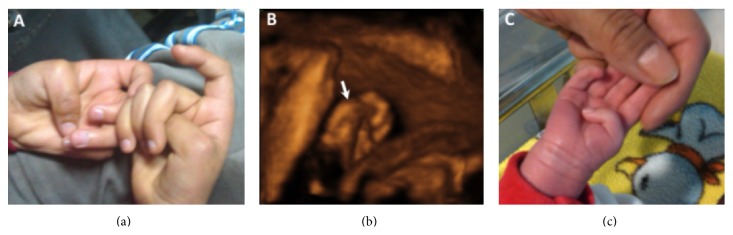
Case 1. (a) Picture of the hands in the index case shows bilateral adducted thumbs. (b) Surface-rendered three-dimensional ultrasound view shows adducted thumb in the fetus at 30 weeks (arrow). (c) Picture of the newborn infant shows adducted thumb.

A 35-year-old woman, gravida 2 para 1, was referred at 30 weeks of gestation due to sonographic detection of fetal hydrocephaly. Her previous history was significant for a son with congenital hydrocephaly, severe neurodevelopment delay, and bilateral adduction of the thumbs (Figure 1(a)). Ultrasound examination at referral confirmed a male infant with bilateral dilatation of the cerebral posterior horns of 25 mm, consistent with severe hydrocephaly. Further examination with 3D ultrasound revealed fixed adduction of the thumbs (Figure 1(b)). With parental consent, cordocentesis was performed for chromosomal analysis. In consideration of the medical history, a fetal sample and peripheral blood from the mother and previous infant were sent for molecular genetic analysis [6]. The screening of the entire coding region of the L1CAM gene (28 exons) led to the identification of a missense mutation (c.803G>A, p.Gly268Asp) in exon 7 of the L1CAM gene in all three samples. At 39 weeks, a 3,500 g male with hydrocephaly and bilateral adduction of the thumbs was delivered by cesarean section (Figure 1(c)). A ventriculoperitoneal shunt was inserted on day 8. However, the infant died at two month of age due to postoperative complications.

## 3. Case 2

**Figure 2:**
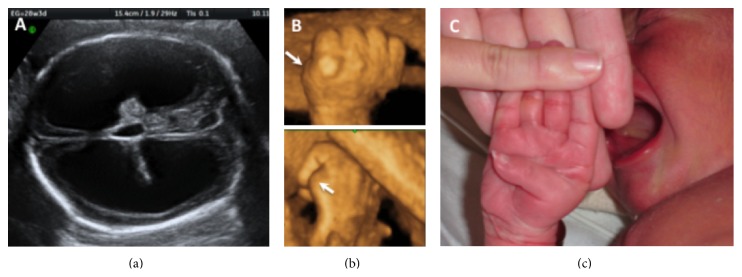
Case 2. (a) Two-dimensional ultrasound view shows severe triventricular hydrocephaly at 28 weeks. (b) Upper and lower panel, surface-rendered three-dimensional ultrasound view shows adducted thumb in the fetus (arrows). (c) Picture of the newborn infant shows adducted thumb.

A 25-year-old woman, gravida 2 para 1, was referred at 28 weeks of gestation due to sonographic detection of fetal hydrocephaly at 25 weeks. Her family and medical histories were unremarkable and her previous pregnancy resulted in a term delivery of a healthy male. Upon consultation, ultrasound examination confirmed a male infant with bilateral dilatation of the cerebral posterior horns of 32 mm, consistent with severe hydrocephaly (Figure 2(a)). Further examination with 3D ultrasound revealed fixed adduction of the thumbs (Figure 2(b)). With parental consent, cordocentesis was performed for cytogenetic and molecular analyses. Maternal blood was also sent for molecular genetic analysis. Direct sequencing on genomic DNA of the 28 exons of the L1CAM gene in maternal and fetal samples was positive for a novel nonsense mutation (c.3241C>T, p.Gln1081X) in exon 24 of the L1CAM gene. At 38 weeks, a 3,285 g male infant with hydrocephaly and bilateral adduction of the thumbs (Figure 1(c)) was delivered by cesarean section. A ventriculoperitoneal shunt was inserted on day 10. The infant is currently 3 years of age; he had not reached minimal neurodevelopmental milestones and is under intensive rehabilitation.

## 4. Discussion

This report documents two cases of X-linked hydrocephaly diagnosed prenatally with 3D ultrasound and confirmed by molecular genetic analysis.  Table 1 shows the relevant molecular genetic analyses in our two cases; the L1CAM gene has missense and nonsense mutations in the exons 7 and 24, respectively. Both cases were referred due to severe triventricular hydrocephaly detected by conventional two-dimensional ultrasound, suggesting a diagnosis of aqueductal stenosis. At referral, 3D ultrasound demonstrated adducted thumbs in both male fetuses, strongly suggesting the diagnosis of X-linked hydrocephaly, HSAS type. In the first case, significant past medical history prompted examination of the fetal hands with 3D ultrasound, demonstrating the abnormal position of the thumbs in the fetus. In the second case, the adducted thumbs were detected as part of the study of congenital hydrocephaly in an index case.

Adducted thumbs are a rare condition that can be associated with a number of genetic syndromes [7]. In a recently published review of 25 pediatric patients with adducted thumbs, only 3 (12%) were isolated findings. Of the remaining 22, 16 were associated with congenital hydrocephaly, of which 4 (25%) were L1CAM syndrome. In the 2 cases presented here, adducted thumbs in the setting of severe hydrocephaly were detected prenatally with 3D ultrasound and the L1CAM mutation was confirmed by molecular genetic analysis. Although our cases were detected in the third trimester due to late referral, prenatal identification of adducted thumbs in a high-risk family has been previously described in three cases, one in the second trimester [8] and two in the first trimester [9]. Our second case seems to be the first in which the fetal hand anomaly was detected prenatally without suggestive medical history.

Our findings may have important clinical implications, considering that examination of the hands is not currently part of the ultrasound protocol in fetuses with severe hydrocephaly. Our investigation supports the examination of fetal hands for adducted thumbs in these cases in order to determine if this is an isolated finding or associated with X-linked aqueductal stenosis. While isolated hydrocephaly poses a very low risk of recurrence, cases of X-linked congenital hydrocephaly have a 50% recurrence risk in male infants. If abnormal positioning of the fetal thumbs is detected in a fetus with severe hydrocephaly, these pregnancies should be eligible for prenatal molecular testing in order to determine the integrity of the L1CAM gene. While examination of the fetal hands has been recommended in the second and third trimesters, as either routine anatomical survey or analysis of potential chromosomal abnormalities or skeletal dysplasias [10], prenatal screening for adducted thumb in the setting of fetal hydrocephaly has not been conducted.

In summary, this report suggests that prenatal ultrasound examination of the fetal hands should be an integral part of the evaluation of fetuses in which hydrocephaly is detected* in utero*. If adducted thumbs are present, molecular studies of the L1CAM gene should be strongly considered. If an L1CAM mutation is confirmed, extensive genetic counseling for the entire family should be provided.

## Figures and Tables

**Table 1:** Genetic information of the mutation detected in our cases.

	Case 1 Mother	Case 1 Fetus	Case 1 Previous son	Case 2 Mother	Case 2 Fetus
Gene	L1CAM	L1CAM	L1CAM	L1CAM	L1CAM
Exon	7	7	7	24	24
Mutation	Missense	Missense	Missense	Nonsense	Nonsense
DNA base	c.803G>A	c.803G>A	c.803G>A	c.3241C>T	c.3241C>T
Aminoacid	p.Gly268Asp	p.Gly268Asp	p.Gly268Asp	p.Gin1081X	p.Gin1081X
